# Genetic predisposition to acute kidney injury – a systematic review

**DOI:** 10.1186/s12882-015-0190-6

**Published:** 2015-12-02

**Authors:** Laura M. Vilander, Mari A. Kaunisto, Ville Pettilä

**Affiliations:** Intensive Care Medicine, University of Helsinki and Helsinki University Hospital, Helsinki, Finland; Institute for Molecular Medicine Finland (FIMM), University of Helsinki, Helsinki, Finland; Folkhälsan Institute of Genetics, Folkhälsan Research Center, Helsinki, Finland

**Keywords:** Acute kidney injury, Genetic variation, Genetic polymorphism

## Abstract

**Background:**

The risk of an individual to develop an acute kidney injury (AKI), or its severity, cannot be reliably predicted by common clinical risk factors. Whether genetic risk factors have an explanatory role poses an interesting question, however. Thus, we conducted a systematic literature review regarding genetic predisposition to AKI or outcome of AKI patients.

**Methods:**

We searched Ovid SP (MEDLINE) and EMBASE databases and found 4027 references to AKI. Based on titles and abstracts, we approved 37 articles for further analysis. Nine were published only as abstracts, leaving 28 original articles in the final analysis. We extracted the first author, year of publication, study design, clinical setting, number of studied patients, patients with AKI, ethnicity of patients, studied polymorphisms, endpoints, AKI definition, phenotype, significant findings, and data for quality scoring from each article. We summarized the findings and scored the quality of articles.

**Results:**

The articles were quite heterogeneous and of moderate quality (mean 6.4 of 10).

**Conclusions:**

Despite different gene polymorphisms with suggested associations with development or severity or outcome of AKI, definitive conclusions would require replication of associations in independent cohort studies and, preferably a hypothesis-free study design.

**Electronic supplementary material:**

The online version of this article (doi:10.1186/s12882-015-0190-6) contains supplementary material, which is available to authorized users.

## Background

Approximately 40 % of critically ill patients develop acute kidney injury (AKI) [1, 2]. Despite other underlying conditions, such as sepsis or circulatory collapse, AKI is independently associated with longer hospital stay and increased mortality [3]. The most recent classification of AKI, namely the KDIGO (Kidney Disease: Improving Global Outcomes) [4] still uses serum-creatinine and urine-output measurements to assess kidney function.

No reliable method exists that is able to predict the individual risk to develop, or to survive, AKI. Genetic variability offers an intriguing research focus, since clinical risk factors explain only part of the conferred risk [5]. Other complex disease states have been investigated for their genetic etiology with promising results, including Crohn’s disease and type 2 diabetes [6, 7]. However, the odds ratios for the common genetic variants known to associate with such traits have been rather modest, approximately 1.1 for the heterozygote genotypes [8].

Individual susceptibility to develop AKI and to influence disease progression have been studied using candidate gene association studies. The literature has been evaluated in two systematic reviews. Lu and colleagues [9] searched articles until the year 2007, and Cardinal-Fernández and colleagues [10] until 2011. Both concluded that no definitive evidence of association of certain genetic variation with AKI exists so far.

Accordingly, we sought to update the current literature about genetic association to AKI with special emphasis on the quality of the studies.

## Methods

### Literature search and selection of studies

We systematically searched the OvidMedline and Embase databases for relevant articles from the year 2000 until March 5, 2015. The databases were selected according to the recommendation of The Human Genome Epidemiology Network (HuGENet) guidelines [11]. We conducted the search using strategies optimized to each database. We aimed to find articles with interest in genetic variability in acute kidney injury. The search criteria were: Genes OR genotype OR phenotype OR polymorphisms OR allelism OR heredity AND acute kidney failure OR acute kidney injury OR acute kidney insufficiency OR acute renal failure OR acute renal injury OR acute renal insufficiency OR acute nephropathy. We used appropriate abbreviations and word truncations, as well as proximity operators, to make the search more comprehensive. The multitude of the near-synonymous terms used in the search is due to the fairly recent acceptance of ‘acute kidney injury’ as the one term to describe the phenomenon, as well as numerous possible ways to discuss genetic variability. We conducted the search with the assistance of a university library information specialist (Helsinki University Library, Terkko, Finland) in English language on March 5, 2015. More detailed information about performing the search is available in Additional material (Additional files 1 and 2).

**Fig. 1:**
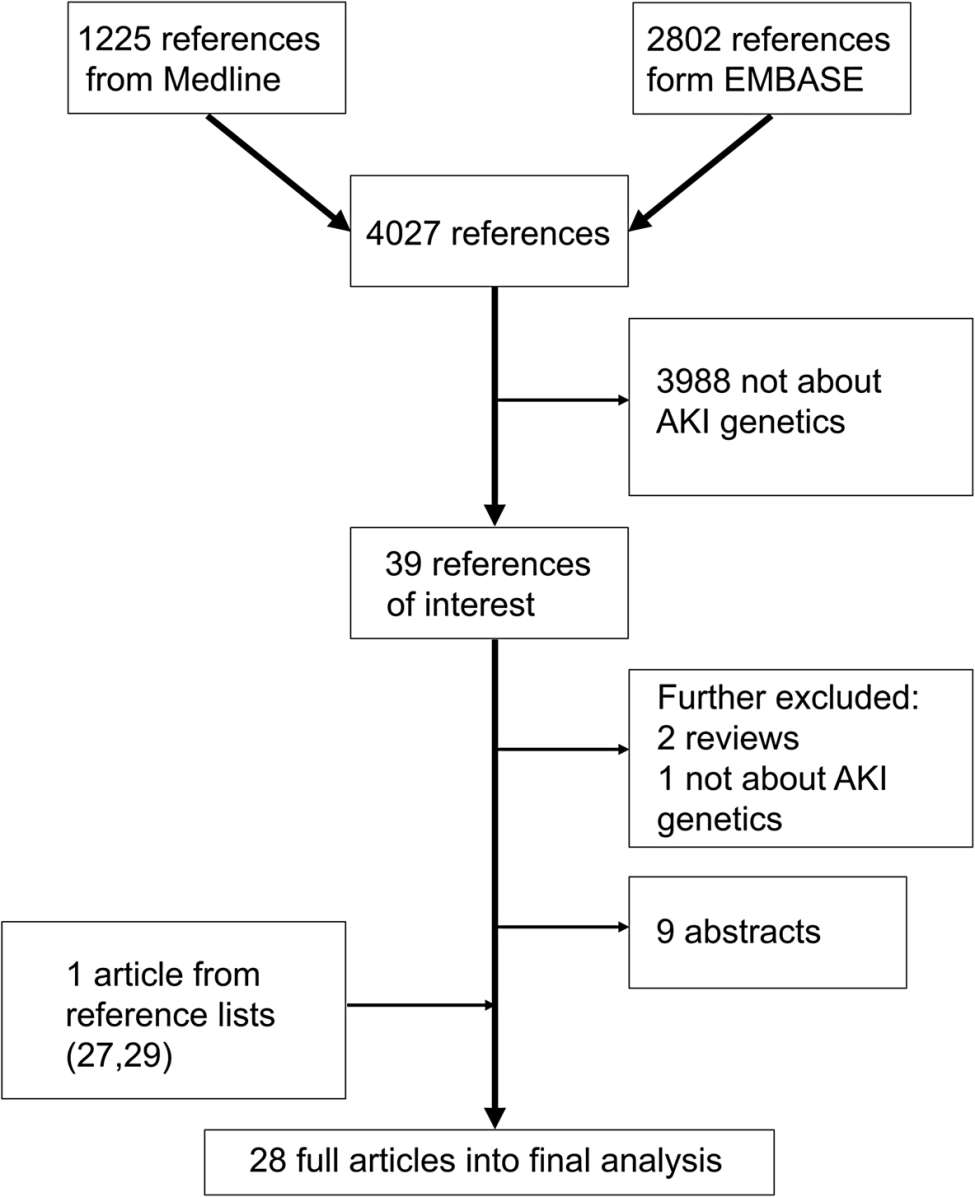
Study flow chart. The literature search was performed on March 5^th^ 2015. Abbreviations: AKI, Acute Kidney Injury

After the removal of duplicates, the search resulted in 4027 references. Articles presenting an original study about human genetic variability and its possible association with AKI were selected for further analysis. We screened titles and abstracts and categorized the articles according to the predefined inclusion and exclusion criteria. Exclusion criteria were determined in an attempt to explore the most common contributors to AKI in adult subjects in a hospital setting. Exclusion was either because of the studied subjects (pediatric patients, organ transplantation patients, experimental animals) or methods used (case studies, review articles). In addition, a number of articles were excluded because they did not study AKI or genetic variability in association with AKI, but focused on chronic renal failure, diabetes, polycystic kidney disease, thrombotic thrombosytopenic purpura, haemolytic uremic syndrome, systemic lupus erythematosus, liver failure, predictive biomarkers, glomerulonephritides, viral infection, malignancies, or hereditary syndromes affecting kidneys. The process of article selection is presented in Fig. 1.

Two authors (L.V and M.K) independently reviewed 39 articles. Of the 39, 9 were published only as conference abstracts [12–20], and two were reviews. One article was about racial difference regarding AKI susceptibility, but failed to explore AKI genetics [21]. We identified one additional article when going through the references of the included articles [22], found it relevant and to fulfil our search criteria and included it according to the HuGENet guidelines [11]. In total, 28 original articles were accepted into the final analysis.

### Data extraction and quality assessment

We extracted the following data from each article: The first author, year of publication, study design, clinical setting, number of all genotyped subjects, number of patients, number of patients with AKI, ethnicity, studied polymorphisms, endpoints, definition of AKI, intermediate phenotype, and significant findings. In addition, we assessed the study quality according to the scoring by Clark et al. [23]. We graded each study on a binary scale of ten quality criteria. One point for each criterion leads to the maximum points of ten. The criteria were predetermined and adapted for better evaluation (Table 1). Studies that were published solely as abstracts [12–17] were not included in this scoring, however some data were extracted and are presented in Additional material (Additional file 3). All correlation calculations were performed using SPSS Statistics version 22 (IBM Corp., Armonk, NY, USA).

**Table 1:** Study quality scoring system adapted from Clark et al. [23]. Studies were assessed using specific criteria described below and scored 1 point if a criterion was fulfilled. [*Adaptation by current reviewers in italic]*

Criterion	Original criteria by Clark et al.	Specific criteria
1. Defining the Control Group	Control group described for size in relation to case group (*n* > case group) and ethnicity, in sufficient detail to allow replication. Cohort studies do not get a point.	*Cohort studies are given a point provided that the cohort is described in sufficient detail, namely the ethnicity of a cohort is given. Prospective cohorts regarded as strong design in demonstrating causality between genetic polymorphism and the development of acute kidney injury (AKI).* ^*a*^ *Size of the control group sufficient when essentially equivalent with case group.*
2. Hardy-Weinberg Equilibrium	Case and control groups assessed for Hardy-Weinberg equilibrium (HWE).	*Cohort or control group should be in HWE (p > 0.01). In case of multiple polymorphisms, the majority should be in HWE.*
3. Defining the Case Group	The disease state of interest as well as inclusion and exclusion criteria of the case group defined in sufficient detail to allow replication.	*Studies that aim to investigate predisposition to AKI are given a point only if they describe the phenotype.*
4. Primer Sequence	The priming sequences used for genotyping or references to them provided.	*Whenever a commercial assay was used for genotyping, a point was given as well.*
5. Describing the Methods	Genotyping method described in sufficient detail to allow replication. In addition, second validating assay performed or the accuracy of the assay validated.	*Accepted quality control methods: duplicated samples or control samples included, second independent investigator analyzing the genotype calls, repeating of ambiguous results.*
6. Blinding	Genotyping performed blinded to clinical status.	
7. Power Calculation	Prospective or retrospective power calculation performed.	*No retrospective power calculations were conducted by the reviewers.*
8. Statistics	Tests of significance presented: such as p values, confidence intervals, or odds ratios.	
9. Corrected Statistics	Corrected for the increased risk of false-positive error in the case of multiple polymorphisms studied. Should *X*2 or odds ratio be used, Hardy-Weinberg equilibrium is to be assessed.	*Where there was only one polymorphism studied, a point was given.*
10. Replication	Study performing second, confirmatory study or confirming earlier polymorphism study.	*A point was given only for studies performing exact replication of a polymorphism in association with a phenotype.*

^*a*^In accordance with Lu et al. [9]

## Results

### Characteristics of articles

The 28 articles in the final analysis were published between 2000 and 2014. The characteristics of the articles are described in detail in Table 2.

**Table 2:** Characteristics of articles

Characteristic	N	References
Clinical setting		
Post cardiac surgery	12	[24, 25, 36–39, 42–44, 46–48]
ICU treatment	6	[26, 33, 34, 40, 41, 49]
Hospitalization with kidney injury	5	[22, 28–31]
Dialysis	1	[27]
Other	4	[32, 35, 45, 50]
Study design		
Prospective cohort	23	[22, 24–30, 32, 33, 36–40, 42–44, 46–50]
Prospective case–control	4	[31, 35, 41, 45]
Retrospective case-cohort	1	[34]
Center of data acquisition		
Single	14	[25, 26, 33, 36, 37, 40, 41, 43–48, 50]
Multi	12	[22, 27–32, 34, 35, 39, 42, 49]
Not applicable	2	[24, 38]
Definition of AKI		
RIFLE	8	[33, 36–40, 42, 46]
AKIN	7	[22, 30–32, 34, 41, 49]
Renal-SOFA	1	[26]
Administration of RRT	2	[44, 47]
Creatinine level	4	[28, 29, 35, 45]
Not applicable	6	[24, 25, 27, 43, 48, 50]
Studied polymorphisms		
1 polymorphism	11	[24, 29, 30, 33, 37, 39, 43, 46–49]
1 gene, several polymorphisms	4	[22, 26, 31, 32]
Several genes	12	[25, 27, 28, 35, 36, 38, 40–42, 44, 45, 50]
GWAS	1	[34]
Ethnicity		
Caucasian	9	[31, 33–36, 40, 46–48]
Mixed	10	[22, 24, 25, 27–30, 32, 49, 50]
Not applicable	9	[26, 37–39, 41–45]
Main outcome		
AKI	26	[22, 25–34, 36–50]
Other	2	[24, 35]

*Abbreviations*: *AKI* Acute Kidney Injury, *AKIN* Acute Kidney Injury Network -classification, *GWAS* Genome-Wide Association Study, *ICU* Intensive Care Unit, *RIFLE* Risk Injury Failure Loss of function End stage -classification, *RRT* renal replacement therapy, *SOFA*, Sequential Organ Failure Assessment score

The number of the patients studied varied between 61 and 1741 (median 262) with a total of 12272. The number of patients with AKI was between 2 and 459 (median 115), but not stated in three studies [24–26]. In six studies [22, 27–31] all genotyped subjects had AKI, in addition to a cohort within one study [32], which comprised only AKI patients. The reviewed articles were grouped into those studying AKI susceptibility and those studying AKI-related outcome. The data extracted from the reviewed articles are presented as Additional file 4 for AKI susceptibility studies and Additional file 5 for AKI related outcome studies.

### Quality of the articles

The mean quality score was 6.4 (range 3–10). (Please see additional file 6). All the studies fulfilled the requirement of reporting basic statistical significance. The AKI phenotype, along with inclusion and exclusion criteria, was adequately defined in 25/28 (89 %) studies. All but two (*N* = 26, 93 %) of the studies provided adequate information about the primer sequences of the genotyping assay.

Only six of 28 (21 %) of the studies reported power calculations [24, 33–37] and only two disclosed the odds ratios [34, 35]. In addition, two of these six studies had some other primary end point than the development or outcome of AKI [24, 35]. Ten of the studies [25, 30, 35–42] could be regarded as replication attempts, since they included at least one polymorphism previously reported to be associated with AKI or related traits. Furthermore, in two (7 %) of the studies [32, 34] showing evidence of association, the result was confirmed in an independent patient sample.

**Fig. 2:**
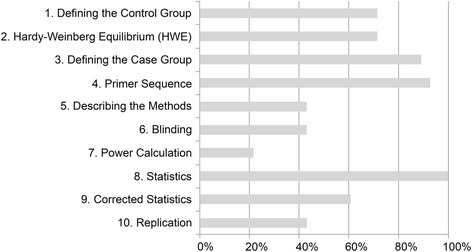
Quality scores according to each criterion. (Full description of criteria is given in Table 1)

The correlation between the two reviewers before consensus was good (Spearman’s rho coefficient 0.90). The mean score for each of the ten quality criteria is presented in Fig. 2.

Of the 28 studies in our review, six (21 %) [24, 25, 27, 28, 38, 43] had been scored previously by Lu [9] (scored 6.0 in this study [range 4–8] vs. Lu et al. 6.3 [range 4–8]), (Spearman’s rho coefficient 0.81).

### Genes associated with AKI

**Fig. 3:**
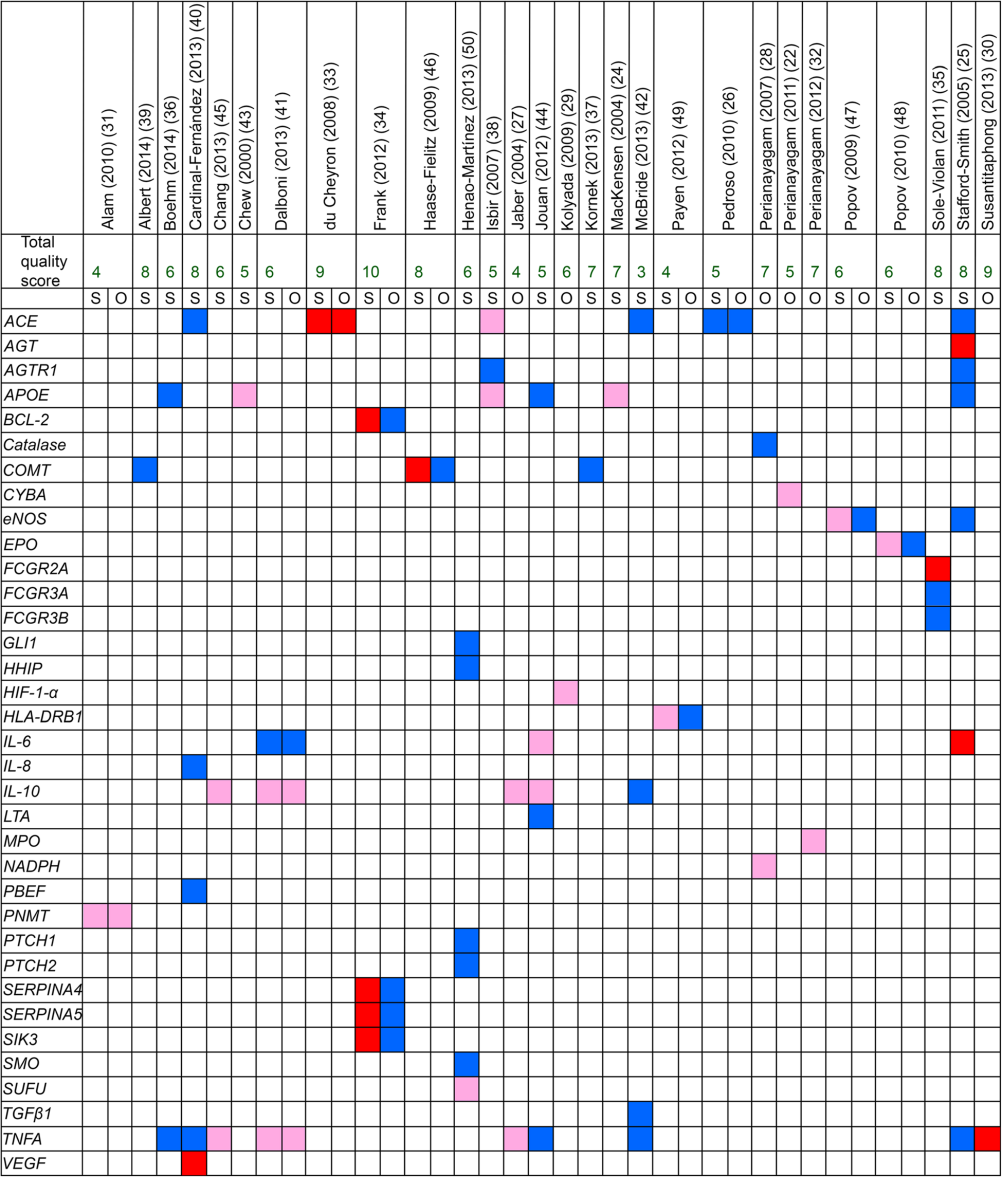
Genes studied for association with either susceptibility to AKI (S) or AKI outcome (O). Red color indicates positive association signals (based on the interpretation of the authors themselves), where dark red signals higher (≥8) quality score and light red signals lower quality score (3–7). Blue color indicates no association. Articles are presented alphabetically by the fist author and genes are presented in alphabetical order. Abbreviations: *ACE*, Angiotensin Converting Enzyme gene; *AGTR1*, Angiotensin II Receptor, Type 1 gene; *AGT*, Angiotensinogen gene; *APOE*, Apolipoprotein-E gene; *BCL-2*, B-cell CLL/lymphoma 2 gene; *COMT*, Catechol-O-methyltransferase gene; *CYBA,* Cytochrome b_245_, α subunit gene; *eNOS*, endothelial Nitric Oxide Synthase gene; *EPO*, Erythropoietin gene; *FCGR2A*, Receptor IIa of the Fc portion of immunoglobulin G gene; *FCGR3A*, Receptor IIIa of the Fc portion of immunoglobulin G gene; *FCGR3B*, Receptor IIIb of the Fc portion of immunoglobulin G gene; *GLI1*, Gli family zinc finger 1 gene; *HHIP*, Hedgehog Interacting Protein gene; *HIF-1-alpha*, Hypoxia-Inducible Factor 1-alpha gene; *HLA-DRB1*, Human Leukocyte Antigen – Major Histocompatibility Complex, Class II, DR beta 1 gene; *IL-6*, Interleukin-6 gene; *IL-8*, Interleukin-8; *IL-10*, Interleukin-10 gene; *LTA*, Lymphotoxin Alpha gene; *MPO*, Myeloperoxidase gene; *NADPH*, Nicotinamide Adenosine Dinucleotide Phosphate gene; *PBEF*, Pre-B cell colony-enhancing factor gene; *PNMT*, Phenylethanolamine N-methyltransferase gene; *PTCH1*, Patched homolog 1 gene; *PTCH2*, Patched homolog 2 gene; *SERPINA4*, Serpin Peptidase Inhibitor, Clade A (alpha-1 antiproteinase, antitrypsin) Member 4 gene; *SERPINA5*, Serpin Peptidase Inhibitor, Clade A (alpha-1 antiproteinase, antitrypsin) Member 5 gene; *SIK3*, Salt-Inducible Kinase 3 gene; *SMO*, Smoothened gene; *SUFU*, Suppressor of Fused homolog gene; *TGF-β*, Transient Growth Factor beta gene; *TNFA*, Tumor Necrosis Factor alpha gene; *VEGF*, Vascular Endothelial Growth Factor gene

The main results are presented in Fig. 3, which lists the investigated genes. Colour coding shows whether the result has been positive or negative and whether the association was found with susceptibility to AKI or its outcome. For more information about the studied polymorphisms and the negative findings, please see Additional files 4 and 5.

### Inflammatory genes

Jouan et al. [44] could not confirm any association between *interleukin 6 (IL-6)* gene −572 G/C polymorphism (rs1800796) or *interleukin 10 (IL-10)* gene −592 C/A polymorphism (rs1800872) and acute renal insufficiency. Stafford-Smith et al. [25] found that a combination of *angiotensinogen (AGT)* gene +842C –allele (rs699) and *IL-6*–572C –allele in Caucasians is associated with renal dysfunction.

The high-producer genotype AA + GA of *tumor necrosis factor alpha (TNFA)* gene −308 G/A polymorphism (rs1800629) was found by Jaber et al. to be associated with higher levels of TNF-α *ex vivo* and higher mortality in patients receiving renal replacement therapy (RRT) [27]. They also demonstrated that *IL-10* polymorphism −1082 G/A intermediate/high producer (GA + GG) genotype (rs1800896) was associated with higher levels of IL-10 and lower mortality in the same patient group [27]. Chang et al. [45] found that in patients undergoing percutaneous coronary intervention (PCI), the A-allele of *TNFA* gene −308 polymorphism, as well as the G-allele of *IL-10*–1082 polymorphism were more prevalent among patients with contrast-induced acute kidney injury. Furthermore, the low-producing genotypes of the aforementioned polymorphisms were associated with the risk of AKI, as were the haplotypes T-C-A of *TNFA* and C-A-C-G of *IL-10*. Dalboni et al. [41] showed that the GG genotype of *TNFA −*308 polymorphism and AA genotype of *IL-10*–1082 polymorphism, when combined, are associated with AKI and/or death, and also with RRT and/or death. Susantitaphong et al. [30] found that *TNFA* gene −308 G/A A-allele was associated with higher peak serum creatinine and urinary kidney injury molecule-1 (KIM-1), as well as higher multi organ failure (MOF) score in hospitalized patients with AKI. The *TNFA* gene was the most investigated, with both positive and negative results (Fig. 3).

In a study by Sole-Violan et al. [35], an association was found between homozygosity for *receptor IIa of the Fc portion of immunoglobulin G (FCGR2A-H131)* allele in the rs1801274 polymorphism and development of acute renal failure in patients with pneumococcal community-associated pneumonia.

### Vasomotor regulation genes

Six studies investigated *angiotensin converting enzyme* (*ACE)* insertion/deletion (I/D) polymorphism (rs4646994) [25, 26, 33, 38, 40, 42]. Isbir et al. [38] were able to find a significant association between *ACE* D–allele and an increased risk of postoperative AKI after coronary artery bypass grafting (CABG). Contradictory to this, du Cheyron et al. [33] found that I/I genotype is associated with an increased risk for AKI and administration of RRT. The remaining studies failed to detect association either in the intensive care unit [26, 40] or after cardiac surgery [25, 42].

Haase-Fielitz [46] studied a low activity (L) Val158Met polymorphism (rs4680) within the *catechol-O-methyltransferase (COMT)* gene. The low-activity genotype (LL) was associated with AKI and more frequent administration of furosemide and RRT. However, results by Albert et al. [39] and Kornek et al. [37] suggest no association between this *COMT* polymorphism and AKI. Association between the C-allele of the *endothelial NO synthase (eNOS)* -786 T/C polymorphism (rs2070744), low creatinine clearance, and more frequent administration of RRT has also been reported [47]. Alam et al. [31] found that *phenylethanolamine N-methyltransferase (PNMT)* gene rs5638 + 1543 G –allele is associated with an increased risk for AKI, and the genotype +1543 G/A is associated with oliguria, whereas *PNMT* rs876493 -161 A –allele is associated with lower mortality and less circulatory shock.

### Other genes

The non-e4-allele of polymorphisms rs429358 and rs7412 of *apolipoprotein E (APOE)* have appeared to associate with a higher maximum creatinine [24, 43], and with increased risk of postoperative AKI [38], in two studies unable to find any association in this post-CABG setting [36, 44]. A study by Kolyada [29] showed that the T-allele of an rs11549465 polymorphism +85 C/T on transcription factor *hypoxia-inducible factor-1alpha (HIF-1α)* gene is associated with higher use of dialysis.

In 2007, Perianayagam and colleagues found a +242 C/T polymorphism (rs4673) in the pro-oxidant enzyme *NADPH (Nicotinamide Adenosine Dinucleotide Phosphate) oxidase p22phox* gene T-allele, associated with more prolonged length of hospital stay, higher dialysis use, and higher odds for composite outcome [28].

In 2012, Perianayagam et al. [32] found an association in a primary cohort of AKI patients between the *myeloperoxidase (MPO)* gene −765 T/C (rs2243828), +9890 A/C (rs2071409), and +2149 T/C (rs2759) polymorphisms and lower urine output as well as the composite outcome of dialysis administration or in-hospital death, and with *MPO +*157 G/T polymorphism (rs7208693) and more advanced AKI as well as the aforementioned composite outcome.

*Cytochrome b*_*245*_*, α subunit (CYBA)* gene, was investigated in a study by Perianayagam et al. [22] who found that rs8854 A-allele was associated with less dialysis requirement or in-hospital death compared with the GG genotype. The haplotype A-A-G-G of polymorphisms rs4782390, rs4673, rs3794624, and rs8854 associated with an increased risk of this outcome.

Popov found the *erythropoietin (EPO)* gene rs1617640 T/G -polymorphism TT-genotype to be associated with increased creatine phosphokinase-MB (CPK-MB) and administration of RRT, but no association with AKI was found [48].

Payen and colleagues [49] found an association between the presence of 4 *human leukocyte antigen-DR-beta (HLA-DRB)* alleles and less administration of RRT, but not with development of AKI.

In a study by Henao-Martínez et al., [50] the polymorphisms rs10786691, rs12414407, rs10748825, and rs7078511 in the *suppressor of fused homolog (SUFU)* gene, correlated with renal function in hospitalized patients with *Enterobacteriacea* sepsis. *Vascular endothelial growth factor (VEGF)* gene polymorphism +936 (rs3025039) CC-genotype was found associated with AKI by Cardinal-Fernández [40].

### Associations identified by a hypothesis-free study design

In a study by Frank [34], two polymorphisms (rs8094315 and rs12457893) within a *B-cell CLL/lymphoma 2 (BCL2)* gene and a single polymorphism (rs2093266) in a *serpin peptidase inhibitor, clade A (alpha-1 antiproteinase, antitrypsin) member 4 (SERPINA4)* gene were found to protect from AKI, and a polymorphism (rs625145) in *salt-inducible kinase 3 (SIK3)* gene was found to be associated with increased risk for AKI.

Polymorphisms studied for their association with the development of AKI and published only as abstracts are summarized in Additional file 3.

## Discussion

In this systematic review we identified 28 studies examining genetic predisposition to the development and/or outcome of AKI. Based on a previously-published quality-scoring method utilized and further developed in this review, these few identified studies had inadequate quality (mean score 6.4/10). The studies were heterogeneous in defining concepts and outcomes, replicated with ambiguous results, and underpowered. All but one of the reviewed studies used a candidate gene approach. Typically, associations found with such an approach have been difficult to replicate and the coverage of genetic variation has been poor [51]. Regardless of some reported positive associations [22, 24, 25, 27–35, 38, 40, 41, 44–50], no conclusive data on predisposing genetic variants exist.

Some generalizations may be made based on the study designs, however. First, the balance between pro- and anti-inflammatory mediators was among the most investigated. Polymorphism rs1800629 in the *TNFA* gene is suspected to alter the inflammatory response and to be associated with AKI susceptibility [41, 45], as well as outcome [27, 30, 41]. This finding is controversial, however; in five studies [25, 36, 40, 42, 44] no evidence supported an association between *TNFA* and susceptibility or outcome of AKI. Second, polymorphisms rs429358 and rs7412 in the *APOE*–gene have been investigated among patients undergoing cardiac surgery. Apolipoprotein E is a protein that is known to function in lipid metabolism but has other roles as well. The *APOE*-e4–allele is suggested to be protective against AKI [24, 38, 43], as is the case with chronic kidney disease [52]. The inverse is true for atherosclerosis and Alzheimer’s disease [53, 54]. Third, renal hemodynamics was one common phenotype of interest in the studies. Consequently, *ACE*, providing a key enzyme in the renin-angiotensin-aldosterone–pathway, with an effect on renal blood flow, is a relevant candidate gene and has been investigated in several studies [25, 26, 33, 38, 40, 42]. Unfortunately, the described associations in the two studies were in opposite directions [33, 38] and the other studies [25, 26, 40, 42] could not confirm any association.

Within the assessed studies, the best-fulfilled quality criteria were: reporting statistics, reporting primer sequence, and defining the case group (Fig. 2). Study statistics were considered adequate if either p-value, confidence intervals, or odds ratios were mentioned. It could be argued that at least the p-value and confidence interval should both be reported in order to correctly evaluate significance of an association. The quality criteria that were most rarely fulfilled were power calculation results, replication, method description, and blinding of clinical status.

Although the initial consensus between the two reviewers was good in this study, the reviewers had several discussions about the specific quality criteria during the process. In general, the original criteria in the reviewed studies were rather subjective and, thus, hard to interpret. Furthermore, the drawback of using the criteria by Clark and colleagues [23] was that these criteria are not perfectly suited for modern genetic study-design or high-throughput genotyping methods. Based on these criteria, points are given relatively easily. More recently, the high probability of spurious, false-positive association signals due to small sample sizes has been better recognized. Thus, we suggest that when assessing the quality of a genetic association study, the most important factors to consider are the sample size and internal replication. Additionally, if the aim is to study the association between a certain gene and a disease/dysfunction, it is crucial that the whole gene should be covered instead of genotyping some random SNPs (Single Nucleotide Polymorphism).

The study by Frank and colleagues [34] avoided most of the weaknesses of the previous studies. Theirs was the first AKI study to employ large-scale genotyping. The quality of this work was superior in many regards compared to other studies discussed in this systematic literature review. They also used an internal replication approach and divided the patients randomly into discovery and validation cohorts. However, their study design was not truly hypothesis-free since a large proportion of the genome was not covered by the genotyping chip used. Of the 142 SNPs showing evidence of association in the discovery cohort, only five could be replicated in the validation cohort. These SNPs were located within the *BCL2*, *SIK3,* and *SERPINA4-SERPINA5* genes, suggesting that apoptotic pathways might have a role in determining the susceptibility to AKI.

The inferences based on this review are subject to some limitations. First, the literature search was conducted in the English language. However, the databases used were selected on the basis of HuGENet guidelines, with assistance from an information specialist. Second, no unpublished data was included in the search. Third, all the possible genetic analyses made by the researchers may not have been published in the articles and, thus, may have led to reporting or publication bias. This may also influence the understanding of the relationship between positive and negative results. Fourth, data proved to be inadequate to allow any meta-analysis. Thus, our findings are limited to the assessment of the quality and description of the individual studies. However, 22 (79 %) and 16 (57 %) of the 28 studies in this review were not included in the two respective previous reviews [9, 10], an obvious strength supporting the additional value of our report.

## Conclusions

In this systematic review we found 28 genetic studies focusing on association with development and/or severity of AKI. Of note, the majority of these studies lacked adequate quality. Conclusive evidence would still require a standard definition of AKI, unbiased outcome measures and replication of associations in a large independent sample and, preferably, applying a genome-wide hypothesis-free study design in future research. This systematic review was reported according to the principles in the Preferred Reporting Items for Systematic Reviews and Meta-Analyses (PRISMA) statement (Additional file 7) [55].

## Additional files

Additional file 1:
**Search strategy in Ovid Medline.** (PDF 32 kb) Additional file 2:
**Search strategy in Embase.** (PDF 33 kb) Additional file 3:
**Studies published only as abstracts.** (PDF 205 kb) Additional file 4:
**Articles studying AKI susceptibility.** (PDF 243 kb) Additional file 5:
**Articles studying AKI-related outcome.** (PDF 213 kb) Additional file 6:
**Quality scoring of the 28 full articles.** (PDF 129 kb) Additional file 7:
**PRISMA Checklist for systematic review.** (PDF 117 kb)
