# Supplementary material for: Genetic predisposition to acute kidney injury – a systematic review
Source: BMC Nephrol. 2015 Dec 2;16:197. doi: 10.1186/s12882-015-0190-6 (PMC4667497; doi:10.1186/s12882-015-0190-6)
Supplement: Additional file 1: — Search strategy in Ovid Medline. (PDF 32 kb) [file 12882_2015_190_MOESM1_ESM.pdf]

Additional file 1. Search Strategy in Ovid Medline, March 5th 2015

1 exp Acute Kidney Injury/

2 (acute adj2 kidney* adj2 failur*).mp

3 (acute adj2 kidney* adj2 injur*).mp

4 (acute adj2 kidney* adj2 insufficien*).mp

5 (acute adj2 renal* adj2 failur*).mp

6 (acute adj2 renal* adj2 injur*).mp

7 (acute adj2 renal* adj2 insufficien*).mp

8 aki.mp

9 (arf and (renal* or kidney*)).mp

10 (acute adj2 nephropath*).mp

11 (acute adj2 nefropath*).mp

12 exp Genetics/

13 exp Genetic Phenomena/

14 (gene or genes* or genet* or hered* or genotyp* or phenotyp* or polymorph* or allelis*).mp,fs

15 1 or 2 or 3 or 4 or 5 or 6 or 7 or 8 or 9 or 10 or 11

16 12 or 13 or 14

17 15 and 16

18 limit to humans

19 limit to years 2000-present
