# Supplementary material for: Genetic predisposition to acute kidney injury – a systematic review
Source: BMC Nephrol. 2015 Dec 2;16:197. doi: 10.1186/s12882-015-0190-6 (PMC4667497; doi:10.1186/s12882-015-0190-6)
Supplement: Additional file 2: — Search strategy in Embase. (PDF 33 kb) [file 12882_2015_190_MOESM2_ESM.pdf]

Additional file 2. Search strategy in Embase, March 5th 2015

S1

EMB.EXACT.EXPLODE("acute kidney failure")

OR (acute NEAR/2 kidney* NEAR/2 failur*)

OR (acute NEAR/2 kidney* NEAR/2 injur*)

OR (acute NEAR/2 kidney* NEAR/2 insufficien*)

OR (acute NEAR/2 renal* NEAR/2 failur*)

OR (acute NEAR/2 renal* NEAR/2 injur*)

OR (acute NEAR/2 renal* NEAR/2 insufficien*)

OR aki

OR (arf AND (renal* OR kidney*))

OR ((acute NEAR/2 nephropath*)

OR (acute NEAR/2 nefropath*))

S2

EMB.EXACT.EXPLODE("genetics")

OR EMB.EXACT.EXPLODE("heredity")

OR (gene or genes* or genet* or hered* or genotyp* or phenotyp* or polymorph* or allelis*)

S3

(S1 AND S2)

AND (subt.exact("human")

AND pd(20000101-20150305))
