# Supplementary material for: Genetic predisposition to acute kidney injury – a systematic review
Source: BMC Nephrol. 2015 Dec 2;16:197. doi: 10.1186/s12882-015-0190-6 (PMC4667497; doi:10.1186/s12882-015-0190-6)
Supplement: Additional file 3: — Studies published only as abstracts. (PDF 205 kb) [file 12882_2015_190_MOESM3_ESM.pdf]

Additional file 3. Studies published only as abstracts.

First author	Year	Study design	Site, acquisition period	Clinical setting	Polymorphisms studied	Number of patients	Number of patients with AKI	Percentage	Ethnicity	Endpoint studied		Definition of AKI	Significant findings
										Susceptibility	Outcome		
Cardinal-Fernandez, P. [1]	2012	Prospective cohort	Single center, 2005-2008	ICU with severe sepsis or septic shock	*ACE* I/D (rs4646994), *TNF-α* -376 (rs1800750), *TNF-α* -308 (rs1800629), *TNF-α* -238 (rs361525), *IL-8* -251 (rs 4073), *VEGF* +405 (rs 2010963), +936 (rs3025039), *PBEF* -1001 (rs9770242)	139	65	46.76 %	Not specified	RIFLE criteria	Not applicable	RIFLE criteria	*VEGF* +936 CC genotype, *PBEF* -1001 GG genotype -> AKI.
Chew, S. [2]	2012	Prospective cohort	Single center	Post cardiac surgery	*ACE* I/D (rs4646994)	991	491	49.55 %	Chinese, Malay, Indian	25% rise in creatinine within 3 postoperative days	Not applicable	25% rise in creatinine postoperatively	Malay and Indian with D allele vs. Chinese -> AKI postoperatively.
Claessen, B. [3]	2013	Prospective cohort	Not specified	PCI patients	*MKL1* -184 C/T, *CYP3A5* -6986 A/G	1018	52	5.11%	Not specified	Relative increase in serum creatinine of ≥25% or absolute increase in serum creatinine of ≥0.5mg/dl in 48h	Not applicable	Relative increase in serum creatinine of ≥25% or absolute increase in serum creatinine of ≥0.5mg/dl in 48h	MKL1 C-184>T CT and TT associated with reduced risk of AKI, CYP3A5 A-6986>G AG and GG associated with increased risk of AKI.
Frank, A. [4]	2010	Prospective cohort	Single center, 1999-2009, data reported elsewhere	ICU with septic shock	GWAS (887 patients)	1264	627	49.60 %	Not specified	AKIN criteria	Not applicable	AKIN criteria	*BCL-2* variant allele of rs8094315 -> decreased risk of AKI.
Kidir, V. [5]	2013	Prospective case-control	Not specified	Hospitalized patients	*Mn-SOD* Ala-9Val	61	61	100%	Turkish	Not reported	Not applicable	Not defined	Differences in genotypic distribution between patients and controls.
Kowalik, M. [6]	2014	Prospective cohort	Not specified	Post cardio-pulmonary bypass	SNV in *ICAM-1, IL-6, LBP1, CRP, NOD2, MASP2, TNFα, SEL-E, NOS3, TLR4*	492	Not specified		Not specified	RIFLE criteria	Not applicable	RIFLE criteria	A combination of *ICAM-1*-rs5498-GG+*TNFα*-rs1800629-GA has higher risk of AKI.
Lagan, A. [7]	2010	Prospective case-control	Single center	Post cardio-pulmonary bypass	14 SNPs from *TNF, LTA, IL-6, IL-10*	137	Not specified		Not specified	Not reported	Duration of stay in ICU, acute kidney injury	Not defined	No reported associations to AKI.
Meyer, N. [8]	2010	Prospective cohort	Not specified	Critically ill trauma	GWAS	414	72	17.39 %	Caucasian, African American	AKIN criteria within 5 days after trauma	Not applicable	AKIN criteria	No significant findings.
Yang, I. [9]	2011	Prospective case-	Not specified	Bacteremia	35 candidate genes	600	Not specified		Not specified	Not reported	Development of adverse	Not defined	Several of the SNPs (*Casp1, Casp4, Casp12,*

		control									outcomes: acute renal failure by renal SOFA score		*Lamn2, Cdx2, Ipo7*) -> development of infection, adverse outcomes: ALI, ARF, coagulation, renal SOFA score.

Abbreviations: A, Adenine; *ACE*, Angiotensin Converting Enzyme -gene; AKI, Acute Kidney Injury; AKIN, Acute Kidney Injury Network; Ala, Alanine; ALI, Acute Lung Injury; ARF, Acute Renal Failure; *BCL-2*, B-cell CLL/lymphoma 2 -gene; C, Cytosine; *Casp*, Caspase -gene; *Cdx2*, Caudal Type Homeobox 2 -gene; *CYP3A5*, Cytochrome P450 3A5 –gene; *CRP*, C-reactive protein –gene; G, Guanine; GWAS, Genome-Wide Association Study; *ICAM-1,* Intercellular Adhesion Molecule 1 –gene, ICU, Intensive Care Unit; I/D, Insertion/Deletion; *IL-6*, Interleukin-6 -gene; *IL-8*, Interleukin-8 -gene; *IL-10*, Interleukin-10 -gene; *Ipo7*, Importin 7 -gene; *LBP1*, Upstream-binding protein 1 –gene; *LTA*, Lymphotoxin Alpha -gene; MASP2, Mannan-binding Lectin Serine Protease 2 –gene; *MKL1*, Megakaryoblastic Leukemia (Translocation) 1 -gene; *Mn-SOD,* Manganese Superoxide Dismutase –gene; *NOD2*, Nucleotide-binding Oligomerization Domain-containing 2 –gene; *NOS3*, Nitric Oxide Synthase 3 –gene; *PBEF*, Pre-B Cell Colony-Enhancing Factor -gene; RIFLE, Risk Injury Failure Loss of function End stage -classification; rs, RefSNP; *SEL-E*, Selectin E –gene; SNP, Single Nucleotide Polymorphism; SOFA, Sequential Organ Failure Assessment; T, Thymine; *TLR4*, Toll-like receptor 4 –gene; *TNF-α*, Tumor Necrosis Factor alpha -gene; Val, Valine; *VEGF*, Vascular Endothelial Growth Factor -gene.

References

1. Cardinal-Fernandez P, Ferruelo A, El-Assar M, Santiago C, Gomez F, Martin-Pellicer A, Frutos-Vivar F, Penuelas O, Nin N, Esteban A, Lorente JA: **Genetic predisposition to acute kidney injury induced by severe sepsis and septic shock [abstract].** *Intensive care medicine* 2012; **38:**103.

2. Chew S, Lian K: **Association of ace d allele with acute kidney injury in non-chinese patients after cardiac surgery in a multi-ethnic South Asian population [abstract].** *49th ERA-EDTA Congress* 2012; .

3. Claessen BE, Syros G, Mehran R, Sanidas E, Chantziara V, Apostolidou I, Weisz G, Rabbani L, Hooper C, Dangas G: **MKL1 C–184>T and CYP3A5 A–6986>G polymorphisms are associated with contrast induced acute kidney injury after PCI.** Journal of the American College of Cardiology 2013, **61**(10_S).

4. Frank A, Sheu C, Chen F, Thompson B, Christiani D: **Clinical and genetic determinants of acute kidney injury in patients with septic shock.** Am J Respir Crit Care Med 2010, **181**(A6818).

5. Kidir V, Uz E, Yigit A, Altuntas A, Yigit B, Inal S, Uz E, Sezer M, Yilmaz R: **Association Between Manganese Superoxide Dismutase Gene Polymorphism and Acute Kidney Injury. Poster Presentation.** Kidney inter , Suppl 2013, **3**(4):410-417.

6. Kowalik MM, Lango R, Chmara M, Bętlejewski P, Wasąg B, Rogowski J, Limon J: **Acute kidney injury may be associated with specific ICAM-1 and TNFα genes variance.** Appl Cardiopulm Pathophysiol 2014, **18**(Suppl. 1):15-16.

7. Lagan A, Hewitt R, Melley D, Hector L, Pantelidis P, Finney S, Evans T, Quinlan G: **Adverse outcomes from cardiac surgery requiring cardiopulmonary bypass: Influence of polymorphism in genes encoding a panel of cytokines [abstract].** *Am J Respir Crit Care Med* 2010; **181:**.

8. Meyer N, Feng R, Li M, Shashaty M, Gallop R, Localio A, Bellamy S, Kaplan S, Lanken P, Fuchs B, Hakonarson H, Aplenc R, Christie J: **Large scale genotyping in a trauma cohort identifies pathways associated with acute kidney injury following trauma [abstract].** *Am J Respir Crit Care Med* 2010; **181:**.

9. Yang I, Warg L, Alper S, Schwartz D: **Association of polymorphisms in known and novel innate immune genes with gram negative bacteremia [abstract].** *Am J Respir Crit Care Med* 2011; **183:**.
