# Supplementary material for: Genetic predisposition to acute kidney injury – a systematic review
Source: BMC Nephrol. 2015 Dec 2;16:197. doi: 10.1186/s12882-015-0190-6 (PMC4667497; doi:10.1186/s12882-015-0190-6)
Supplement: Additional file 4: — Articles studying AKI susceptibility. (PDF 243 kb) [file 12882_2015_190_MOESM4_ESM.pdf]

Additional file 4. Articles studying AKI susceptibility.

First author	Year	Study design	Site, acquisition period	Clinical setting	Polymorphisms studied	Number of patients	Number of patients with AKI	Percentage	Ethnicity	Endpoint studied	Definition of AKI	Intermediate phenotype association	Total quality score
**Alam, A.** [1]	2010	Prospective case-control	Multi center (2), 2003-2007	Hospitalized with AKI	*PNMT* -161 G/A (rs876493), *PNMT* 1543 A/G (rs5638)	961	194	20.19 %	Caucasian	Increase in serum creatinine at enrollment, according to modified AKIN criteria	AKIN criteria	Urine adrenaline and noradrenaline concentration	4
**Albert, C.** [2]	2014	Prospective cohort	Multi center	Elective cardiac surgery with CPB	*COMT*-Val158Met (rs4680)	195	22	11.28%	Not specified	Postoperative AKI according to the RIFLE criteria	RIFLE criteria	Not specified	8
**Boehm, J.** [3]	2014	Prospective cohort	Single center	Elective cardiac surgery with CPB	*APOE* e2/e3/e4 (rs429358 & rs7412), *TNFA* -308 G/A (rs1800629)	1415	250	17.67%	Caucasian	CSA-AKI according to RIFLE criteria (no Failure group)	RIFLE criteria (without Failure group)	Not specified	6
**Cardinal-Fernández, P.** [4]	2013	Prospective cohort	Single center, 2005-2008	ICU with severe sepsis	*ACE* I/D (rs4646994), *TNFA* -376 G/A (rs1800750), -308 G/A (rs1800629), -238 G/A (rs361525), *IL-8* -251 T/A (rs4073), *VEGF* +405 G/C (rs2010963), +936 C/G (rs3025039), *PBEF* -1001 T/G (rs9770242)	139	65	46.76%	White	R, I or F category according to RIFLE criteria during ICU stay	RIFLE criteria	Not specified	8
**Chang, C.** [5]	2013	Prospective case-control	Single center, 2008-2010	PCI with CM	*TNFA* -308 G/A (rs1800629) + 2 polymorphisms (rs1799964, rs1800630), *IL-10* -1082 G/A (rs1800896) + 3 polymorphisms (rs1554286, rs3021094, rs3790622)	508	53	10.43%	Not specified	CIN, defined as increase in serum Cr of ≥0.5mg/dl or a 25% increase from baseline, within 48h after PCI	Increase in serum Cr of ≥0.5mg/dl or a 25% increase from baseline, within 48h after PCI	Baseline plasma IL-10 and TNF-α	6
**Chew, S.** [6]	2000	Prospective cohort	Single center, 1989-1999	Post-CABG	*APOE* e2/e3/e4 (rs429358 & rs7412)	564	2	0.35 %	Not specified	Change in serum creatinine as continuous variable: Peak serum creatinine, perioperative difference	Not defined	Not specified	5
**Dalboni, M.** [7]	2013	Prospective case-control	Single center	ICU	*TNFA* -308 G/A (rs1800629), *IL-6* -174 G/C (rs1800795), *IL-10* -1082 G/A (rs1800896)	303	139	45.87%	Not specified	According to AKIN and RIFLE criteria	Threefold increase of serum creatinine compared with baseline or creatinine clearance < 60ml/min/mm^3	Not specified	6
**du Cheyron, D.** [8]	2008	Prospective cohort	Single center, 2006	ICU	*ACE* I/D (rs4646994)	180	73	40.56 %	Caucasian	According to RIFLE-criteria during ICU stay	RIFLE criteria	Serum ACE level	9
**Frank, A.** [9]	2012	Retrospective case-cohort	Multi center (2), 1999-2009, 2007-2009	ICU with septic shock	GWAS	887	459	51.75 %	Caucasian	According to AKIN criteria (creatinine) within first 72h of ICU admission	AKIN criteria	Not specified	10

**Haase-Fielitz, A.** [10]	2009	Prospective cohort	Single center	Post cardiopulmonary bypass	*COMT* 472G/A (Val108/158Met, rs4680)	260	53	20.38 %	Caucasian	Increase in serum creatinine >50% in 5 days according to modified RIFLE criteria	RIFLE criteria	Plasma norepinephrine, epinephrine, L-DOPA, DHPG concentrations at baseline, 6, 24 h	8
**Henao-Martínez, A.** [11]	2013	Prospective cohort	Single center, 2002-2007	Hospitalized with *Enterobacteriacea* sepsis	69 SNPs in 6 genes *(PTCH1, PTCH2, GLI1, SMO, HHIP, SUFU* (rs10786691, rs12414407, rs10748825, rs10748827, rs7078511, rs2296590, rs7907760 in *SUFU))*	159	12	7.54%	72% Caucasian, 28% African American,	Creatinine levels as continuous variable and an indicator of renal function and organ injury	Not defined	Not specified	6
**Isbir, S.** [12]	2007	Prospective cohort	2004-2006	Post-CABG	*ACE* I/D (rs4646994), *APOE* e2,e3,e4, (rs429358 & rs7412), *AGTR1* 1166 A/C (rs5186)	248	54	21.77 %	Not specified	According to RIFLE criteria	RIFLE criteria	Plasma level of ACE	5
**Jouan, J.** [13]	2012	Prospective cohort	Single center	Post cardiopulmonary bypass	*LTA* 252 A/G (rs909253), Cys13Arg (rs2229094), *TNF* 308 G/A (rs1800629), *IL6* -597 G/A (rs1800797), 572 G/C (rs1800796), -174 G/C (rs1800795), *IL10* -592 C/A (rs1800872), *117 C/T (rs3024496), *APOE* 388 T/C, 526 C/T (rs429358 & rs7412)	126	8	6.35 %	Not specified	Plasma creatinine level >200mikromol/l or requirement for dialysis at any time after surgery	Plasma creatinine levels greater than 200 mikromol/l, requirement of hemodialysis	Plasma concentrations of IL-6, IL-10 and TNFα	5
**Kornek, M.** [14]	2013	Prospective cohort	Single center, 2006-2009	Cardiac surgery with CPB	*COMT*-Val158Met (rs4680)	1741	398	22.86%	Not specified	Increase in postoperative creatinine and CSA-AKI according to RIFLE criteria	RIFLE criteria	Not specified	7
**MacKensen, G.** [15]	2004	Prospective cohort	1999-2000	Post-CABG	*APOE* e4 (rs429358 & rs7412)	130			Caucasian, African American 4.6%	Change in serum creatinine as continuous variable: Postoperative peak serum creatinine	Not defined	Not specified	7
**McBride, W.** [16]	2013	Prospective cohort	Multi center (2)	Elective cardiac surgery	*TNFA* -308 G/A (rs1800629), *IL-10* -1082 G/A (rs1800896), *ACE I/D* (rs4646994), *TGFβ1* -509 C/T (rs1800469)	408	49	12.01%	Not specified	According to RIFLE criteria, eGFR drop >25%, categorized as early and late renal dysfunction	RIFLE criteria	Baseline and +2h plasma IL-10 and TNF-α; baseline and +24h urinary TGFβ1, IL-1ra and TNFsr2	3
**Payen, D.** [17]	2012	Prospective cohort	Multi center (4), 2004-2005	Severe sepsis or septic shock	*HLA-DRB1*	176	129	73.30 %	Caucasian 94%	According to AKIN value during first 48h	AKIN criteria	Monocyte HLA-DR expression, plasma cytokines (IL-6, IL-10, MIF)	4
**Pedroso, J.** [18]	2010	Prospective cohort	Single center, 2002, 2005	ICU	*ACE* I/D (rs4340), -262 A/T (rs4291)	153		0.00 %	Not specified	Evolution of renal-SOFA score during ICU days 1-7	Renal-SOFA	Not specified	5
**Popov, A.** [19]	2009	Prospective cohort	Single center	Post cardiopulmonary bypass	*eNOS* -786 T/C (rs2070744)	497	161	32.39 %	White	Reduced creatinine clearance, rise in plasma creatinine as continuous variable and postoperative dialysis requirement	Postoperative dialysis requirement	Not specified	6

**Popov, A.** [20]	2010	Prospective cohort	Single center, 2006-2007	Post cardiopulmonary bypass	*EPO* (rs1617640)	481	152	31.60 %	Caucasian	Highest postoperative creatinine and perioperative difference as continuous variables, postoperative dialysis requirement	Not defined	Not specified	6
**Sole-Violan, J.** [21]	2011	Prospective case-control	Multi center (4), 2003-2009	Hospitalized with community acquired pneumonia	*FCGR2A* H131R/H (rs1801274), *FCGR3A* V158F (rs396991), *FCGR3B* NA1/NA2	1262 (319 with PCAP)	101 (with PCAP)	31.66% (in PCAP)	Caucasoid Spanish	Urine output <20ml/h and/or creatinine level >2mg/dl when previously normal	Urine output <20ml/h and/or creatinine level <2mg/dl	Not specified	8
**Stafford-Smith, M.** [22]	2005	Prospective cohort	Single center, 1995-2002	Post-CABG	12 out of which 7 specified: *ACE* I/D (rs4646994), *AGT* 842 T/C (rs699), *AGTR1* 1166 A/C (rs5186), *eNOS* 894 G/T (rs1799983), IL-6 -572 G/C (rs1800796), *TNFA* -308 G/A (rs1800629), *APOE* 448 T/C, 586 C/T (rs429358 & rs7412)	1671		0.00 %	88% Caucasian, 12% African American	Peak fractional change in postoperative serum creatine as continuous variable	Not defined	Not specified	8

Abbreviations: A, Adenine; ACE, Angiotensin Converting Enzyme; *ACE*, Angiotensin Converting Enzyme gene; *AGTR1*, Angiotensin II Receptor, Type 1 gene; *AGT*, Angiotensinogen gene; AKI, Acute Kidney Injury; AKIN, Acute Kidney Injury Network; *APOE*, Apolipoprotein-E gene; Arg, Arginine; bp, Base Pair; C, Cytosine; CABG, Coronary Artery Bypass Grafting; CIN; Contrast-Induced Nephropathy; CM, Contrast Medium; *COMT*, Catechol-O-methyltransferase gene; CPB, Cardiopulmonary Bypass; CSA, Cardiac Surgery Associated; Cys, Cysteine; DHBG, DiHydroxyButylGuanine; eGFR, estimated Glomerular Filtration Rate; *eNOS*, endothelial Nitric Oxide Synthase -gene; *EPO*, Erythropoietin gene; *FCGR2A*, Receptor IIa of the Fc portion of immunoglobulin G gene; *FCGR3A*, Receptor IIIa of the Fc portion of immunoglobulin G gene; *FCGR3B*, Receptor IIIb of the Fc portion of immunoglobulin G gene; G, Guanine; *GLI1*, Gli family zinc finger 1 gene; GWAS, Genome-Wide Association Study; *HHIP*, Hedgehog Interacting Protein gene; HLA-DR, Human Leukocyte Antigen – Major Histocompatibility Complex, Class II, DR; *HLA-DRB1*, Human Leukocyte Antigen – Major Histocompatibility Complex, Class II, DR beta 1 gene; ICU, Intensive Care Unit; I/D, Insertion/Deletion; IgG FC, Immunoglobulin G Fc Fragment; IL-1ra, Interleukin-1 receptor antagonist; IL-6, Interleukin-6; *IL-6*, Interleukin-6 gene; *IL-8*, Interleukin-8 gene; IL-10, Interleukin-10; *IL-10*, Interleukin-10 gene; L-DOPA, Levodopa; LTA, Lymphotoxin Alpha; MIF, Macrophage Migration Inhibitory Factor; Met, Methionine; *PBEF*, pre-B-cell colony-enhancing factor gene; PCAP, Pneumococcal Community-Acquired Pneumonia; PCI, Percutaneous Coronary Intervention; *PNMT*, Phenylethanolamine N-methyltransferase gene; *PTCH1*, Patched homolog 1 gene; *PTCH2*, Patched homolog 2 gene; RIFLE, Risk Injury Failure Loss of function End stage -classification; rs, RefSNP; *SMO*, Smoothened gene; SOFA, Sequential Organ Failure Assessment; T, Thymine; TGFβ1, Transforming Growth Factor beta 1; *TGFβ1*, Transforming Growth Factor beta 1 gene; TNF-α, Tumor Necrosis Factor alpha; TNFsr2, Tumor Necrosis factor alpha soluble receptor p75; *TNFA*, Tumor Necrosis Factor alpha gene; Val, Valine; *VEGF*, Vascular endothelial growth factor gene.

References

1. Alam A, O'Connor DT, Perianayagam MC, Kolyada AY, Chen Y, Rao F, Mahata M, Mahata S, Liangos O, Jaber BL: **Phenylethanolamine N-methyltransferase gene polymorphisms and adverse outcomes in acute kidney injury.** Nephron 2010, **114**(4):253-259.

2. Albert C, Kube J, Haase-Fielitz A, Dittrich A, Schanze D, Zenker M, Kuppe H, Hetzer R, Bellomo R, Mertens PR, Haase M: **Pilot study of association of catechol-O-methyl transferase rs4680 genotypes with acute kidney injury and tubular stress after open heart surgery.** Biomark med 2014, **8**(10):1227-1238.

3. Boehm J, Eichhorn S, Kornek M, Hauner K, Prinzing A, Grammer J, Lahm H, Wagenpfeil S, Lange R: **Apolipoprotein E genotype, TNF-alpha 308G/A and risk for cardiac surgery associated-acute kidney injury in Caucasians.** Ren Fail 2014, **36**(2):237-243.

4. Cardinal-Fernandez P, Ferruelo A, El-Assar M, Santiago C, Gomez-Gallego F, Martin-Pellicer A, Frutos-Vivar F, Penuelas O, Nin N, Esteban A, Lorente JA: **Genetic predisposition to acute kidney injury induced by severe sepsis.** J Crit Care 2013, **28**(4):365-370.

5. Chang CF, Lu TM, Yang WC, Lin SJ, Lin CC, Chung MY: **Gene polymorphisms of interleukin-10 and tumor necrosis factor-alpha are associated with contrast-induced nephropathy.** Am J Nephrol 2013, **37**(2):110-117.

6. Chew ST, Newman MF, White WD, Conlon PJ, Saunders AM, Strittmatter WJ, Landolfo K, Grocott HP, Stafford-Smith M: **Preliminary report on the association of apolipoprotein E polymorphisms, with postoperative peak serum creatinine concentrations in cardiac surgical patients.** Anesthesiology 2000, **93**(2):325-331.

7. Dalboni MA, Quinto BM, Grabulosa CC, Narciso R, Monte JC, Durao M Jr, Rizzo L, Cendoroglo M, Santos OP, Batista MC: **Tumour necrosis factor-alpha plus interleukin-10 low producer phenotype predicts acute kidney injury and death in intensive care unit patients.** Clin Exp Immunol 2013, **173**(2):242-249.

8. du Cheyron D, Fradin S, Ramakers M, Terzi N, Guillotin D, Bouchet B, Daubin C, Charbonneau P: **Angiotensin converting enzyme insertion/deletion genetic polymorphism: its impact on renal function in critically ill patients.** Crit Care Med 2008, **36**(12):3178-3183.

9. Frank AJ, Sheu CC, Zhao Y, Chen F, Su L, Gong MN, Bajwa E, Thompson BT, Christiani DC: **BCL2 genetic variants are associated with acute kidney injury in septic shock*.** Crit Care Med 2012, **40**(7):2116-2123.

10. Haase-Fielitz A, Haase M, Bellomo R, Lambert G, Matalanis G, Story D, Doolan L, Buxton B, Gutteridge G, Luft FC, Schunck WH, Dragun D: **Decreased catecholamine degradation associates with shock and kidney injury after cardiac surgery.** J Am Soc Nephrol 2009, **20**(6):1393-1403.

11. Henao-Martinez AF, Agler AH, LaFlamme D, Schwartz DA, Yang IV: **Polymorphisms in the SUFU gene are associated with organ injury protection and sepsis severity in patients with Enterobacteriacea bacteremia.** Infect Genet Evol 2013, **16**:386-391.

12. Isbir SC, Tekeli A, Ergen A, Yilmaz H, Ak K, Civelek A, Zeybek U, Arsan S: **Genetic polymorphisms contribute to acute kidney injury after coronary artery bypass grafting.** Heart Surg Forum 2007, **10**(6):E439-44.

13. Jouan J, Golmard L, Benhamouda N, Durrleman N, Golmard JL, Ceccaldi R, Trinquart L, Fabiani JN, Tartour E, Jeunemaitre X, Menasche P: **Gene polymorphisms and cytokine plasma levels as predictive factors of complications after cardiopulmonary bypass.** J Thorac Cardiovasc Surg 2012, **144**(2):467; Aug-473.

14. Kornek M, Deutsch MA, Eichhorn S, Lahm H, Wagenpfeil S, Krane M, Lange R, Boehm J: **COMT-Val158Met-polymorphism is not a risk factor for acute kidney injury after cardiac surgery.** Dis Markers 2013, **35**(2):129-134.

15. MacKensen GB. Swaminathan M. Ti LK. Grocott HP. Phillips-Bute BG. Mathew JP. Newman MF. Milano CA. Stafford-Smith M. Perioperative Outcomes Research Group. Cardiothoracic Anesthesiology Research Endeavors (C.A.R.E.) Investigators of the Duke Heart Center: **Preliminary report on the interaction of apolipoprotein E polymorphism with aortic atherosclerosis and acute nephropathy after CABG.** Ann Thorac Surg 2004, **78**(2):520-526.

16. McBride WT, Prasad PS, Armstrong M, Patterson C, Gilliland H, Drain A, Vuylsteke A, Latimer R, Khalil N, Evans A, Cambien F, Young I: **Cytokine phenotype, genotype, and renal outcomes at cardiac surgery.** Cytokine 2013, **61**(1):275-284.

17. Payen D, Lukaszewicz AC, Legrand M, Gayat E, Faivre V, Megarbane B, Azoulay E, Fieux F, Charron D, Loiseau P, Busson M: **A multicentre study of acute kidney injury in severe sepsis and septic shock: association with inflammatory phenotype and HLA genotype.** PLoS ONE 2012, **7**(6):e35838.

18. Pedroso JA, Paskulin D, Dias FS, de Franca E, Alho CS: **Temporal trends in acute renal dysfunction among critically ill patients according to I/D and -262A > T ACE polymorphisms.** J Bras Nefrol 2010, **32**(2):182-194.

19. Popov AF, Hinz J, Schulz EG, Schmitto JD, Wiese CH, Quintel M, Seipelt R, Schoendube FA: **The eNOS 786C/T polymorphism in cardiac surgical patients with cardiopulmonary bypass is associated with renal dysfunction.** Eur J Cardiothorac Surg 2009, **36**(4):651-656.

20. Popov AF, Schulz EG, Schmitto JD, Coskun KO, Tzvetkov MV, Kazmaier S, Zimmermann J, Schondube FA, Quintel M, Hinz J: **Relation between renal dysfunction requiring renal replacement therapy and promoter polymorphism of the erythropoietin gene in cardiac surgery.** Artif Organs 2010, **34**(11):961-968.

21. Sole-Violan J, Garcia-Laorden MI, Marcos-Ramos JA, de Castro FR, Rajas O, Borderias L, Briones ML, Herrera-Ramos E, Blanquer J, Aspa J, Florido Y, Garcia-Bello MA, Ferrer-Aguero JM, Sologuren I, Rodriguez-Gallego C: **The Fc receptor IIA-H/H131 genotype is associated with bacteremia in pneumococcal community-acquired pneumonia.** Crit Care Med 2011, **39**(6):1388-1393.

22. Stafford-Smith M. Podgoreanu M. Swaminathan M. Phillips-Bute B. Mathew JP. Hauser EH. Winn MP. Milano C. Nielsen DM. Smith M. Morris R. Newman MF. Schwinn DA. Perioperative Genetics and Safety Outcomes Study (PEGASUS) Investigative Team: **Association of genetic polymorphisms with risk of renal injury after coronary bypass graft surgery.** Am J Kidney Dis 2005, **45**(3):519-530.
