# Supplementary material for: Genetic predisposition to acute kidney injury – a systematic review
Source: BMC Nephrol. 2015 Dec 2;16:197. doi: 10.1186/s12882-015-0190-6 (PMC4667497; doi:10.1186/s12882-015-0190-6)
Supplement: Additional file 5: — Articles studying AKI-related outcome. (PDF 213 kb) [file 12882_2015_190_MOESM5_ESM.pdf]

Additional file 5. Articles studying AKI related outcome.

First author	Year	Study design	Site, acquisition period	Clinical setting	Polymorphisms studied	Number of patients	Number of patients with AKI	Percentage	Ethnicity	Endpoint studied	Definition of AKI	Intermediate phenotype association	Total quality score
**Alam, A.** [1]	2010	Prospective case-control	Multi center (2), 2003-2007	Hospitalized with AKI	*PNMT* -161 G/A (rs876493), *PNMT* 1543 A/G (rs5638)	961	194	20.19 %	Caucasian	In-hospital mortality, dialysis administration, presence of oliguria	AKIN criteria	Urine adrenaline and noradrenaline concentration	4
**Dalboni, M.** [2]	2013	Prospective case-control	Single center	ICU	*TNFA* -308 G/A (rs1800629), *IL-6* -174 G/C (rs1800795), *IL-10* -1082 G/A (rs1800896)	303	139	45.87%	Not specified	Mortality	Threefold increase of serum creatinine compared with baseline or creatinine clearance < 60ml/min/mm^3	Not specified	6
**du Cheyron, D.** [3]	2008	Prospective cohort	Single center, 2006	ICU	*ACE* I/D (rs4646994)	180	73	40.56 %	Caucasian	Duration of ICU stay, mortality	RIFLE criteria	Serum ACE level	9
**Haase-Fielitz, A.** [4]	2009	Prospective cohort	Single center	Post cardiopulmonary bypass	*COMT* 472G/A (Val108/158Met, rs4680)	260	53	20.38 %	Caucasian	Dialysis administration	RIFLE criteria	Plasma norepinephrine, epinephrine, L-DOPA, DHPG concentrations at baseline, 6, 24 h	8
**Jaber, B.** [5]	2004	Prospective cohort	Multi center (2), 1996-2000	Intermittent hemodialysis	*TNFA* -308 G/A (rs1800629), *IL-10* -1082 G/A (rs1800896)	61	61	100.00 %	93% Caucasian	Mortality, renal recovery (discontinuation of dialysis)	Not defined	Ex vivo production of TNF-[illegible] and IL-10 in a subset of patients	4
**Kolyada, A.** [6]	2009	Prospective cohort	Multi center (3), 2003-2008	Hospitalized with nephrology consultation	*HIF-1α* 85 C/T (rs11549465)	241	241	100.00 %	91% white, 6% black, 3% other	Dialysis administration, in-hospital mortality, composite outcome	Rise in serum creatinine	Plasma levels of VEGF-A and Ang-2	6
**Payen, D.** [7]	2012	Prospective cohort	Multi center (4), 2004-2005	Severe sepsis or septic shock	*HLA-DRB1*	176	129	73.30 %	94% Caucasian	Severity of AKI from mild to severe, 28 day mortality	AKIN criteria	Monocyte HLA-DR expression, plasma cytokines (IL-6, IL-10, MIF)	4
**Pedroso, J.** [8]	2010	Prospective cohort	Single center, 2002, 2005	ICU	*ACE* I/D (rs4340), -262 A/T (rs4291)	153		0.00 %	Not specified	Duration of hospital stay, mortality	Renal-SOFA	Not specified	5
**Perianayagam, M.** [9]	2007	Prospective cohort	Multi center (2), 2003-2006	Hospitalized with ARF	*NADPH Oxidase p22phox* +242 C/T (rs4673), *catalase* -262 C/T (rs1001179)	200	200	100.00 %	90.5% white, 5.5% black, 4% other	Duration of hospital stay, dialysis administration, in-hospital death, composite outcome	Rise in serum creatinine	Nitrotyrosine concentration, catalase activity (measured as dismutation of hydrogen peroxide)	7

**Perianayagam, M.** [10]	2011	Prospective cohort	Multi center (2), 2003-2010	Hospitalized with AKI	*CYBA* -1442 G/A (rs8854), +383 G/A (rs3794624), +242 C/T (rs4673), +8897 A/T (rs4782390), +640 A/G (rs1049255)	262	262	100%	91% white, 7% black, 2% other	Dialysis administration, in-hospital death, composite outcome	AKIN criteria	Nitrotyrosine concentration, urinary levels of 15-F2t-isoprostane	5
**Perianayagam, M.** [11]	2012	Prospective cohort in two different approaches	Multi center (2,4), 2003-2010, 2004-2011	1) hospitalized with AKI, 2) on-pump cardiac surgery	*MPO* -765 T/C (rs2243828), 157 G/T (rs7208693), 9890 A/C (rs2071409), 2149 T/C (rs2759)	1) 262 2) 277	1) 262 2) 7	1) 100% 2) 2.53%	1) 91% white 2) 95% white	Severity of AKI, adverse clinical outcomes (dialysis administration, in-hospital death)	Rise in serum creatinine from baseline	Plasma levels of MPO, urinary levels of 15-F2t-isoprostane	7
**Popov, A.** [12]	2009	Prospective cohort	Single center	Post cardiopulmonary bypass	*eNOS* -786 T/C (rs2070744)	497	161	32.39 %	White	Dialysis administration	Postoperative dialysis requirement	Not specified	6
**Popov, A.** [13]	2010	Prospective cohort	Single center, 2006-2007	Post cardiopulmonary bypass	*EPO* (rs1617640)	481	152	31.60 %	Caucasian	Dialysis administration	Not defined	Not specified	6
**Susantitaphong, P.** [14]	2013	Prospective cohort	Multi center (2), 2003-2007	Hospitalized with AKI	*TNFA* -308 G/A (rs1800629)	262	262	100%	91% white	Severity of AKI, tubular injury demonstrated by urinary NAG, KIM-1, [illegible]-GST, [illegible]-GST	AKIN criteria	Not specified	9

Abbreviations: A, Adenine; *ACE*, Angiotensin Converting Enzyme gene; AKI, Acute Kidney Injury; AKIN, Acute Kidney Injury Network; Ang-2, Angiotensin-2; *APOE*, Apolipoprotein-E gene; ARF, Acute Renal Failure; bp, Base Pair; C, Cytosine; *COMT*, Catechol-O-methyltransferase gene; *CYBA*, Cytochrome b_{245}, α subunit gene; DHBG, DiHydroxyButylGuanine; *eNOS*, endothelial Nitric Oxide Synthase gene; *EPO*, Erythropoietin gene; G, Guanine; GST, Glutathione S-transferase; *HIF-1-α*, Hypoxia-Inducible Factor 1-alpha gene; *HLA-DRB1*, Human Leukocyte Antigen – Major Histocompatibility Complex, Class II, DR beta 1 gene; ICU, Intensive Care Unit; I/D, Insertion/Deletion; *IL-6*, Interleukin-6 gene; *IL-10*, Interleukin-10 gene; KIM-1, Kidney Injury Molecule-1; L-DOPA, Levodopa; Met, Methionine; MPO, Myeloperoxidase; *MPO*, Myeloperoxidase gene; *NADPH*, Nicotinamide Adenosine Dinucleotide Phosphate gene; NAG, N-acetyl-β-D-glucosaminidase; PCAP, Pneumococcal Community-Acquired Pneumonia; *PNMT*, Phenylethanolamine N-methyltransferase gene; RIFLE, Risk Injury Failure Loss of function End stage -classification; rs, RefSNP; SOFA, Sequential Organ Failure Assessment; T, Thymine; *TNFA*, Tumor Necrosis Factor alpha gene; Val, Valine; VEGF, Vascular Endothelial Growth Factor.

References

1. Alam A, O'Connor DT, Perianayagam MC, Kolyada AY, Chen Y, Rao F, Mahata M, Mahata S, Liangos O, Jaber BL: **Phenylethanolamine N-methyltransferase gene polymorphisms and adverse outcomes in acute kidney injury.** Nephron 2010, **114**(4):253-259.

2. Dalboni MA, Quinto BM, Grabulosa CC, Narciso R, Monte JC, Durao M Jr, Rizzo L, Cendoroglo M, Santos OP, Batista MC: **Tumour necrosis factor-alpha plus interleukin-10 low producer phenotype predicts acute kidney injury and death in intensive care unit patients.** Clin Exp Immunol 2013, **173**(2):242-249.

3. du Cheyron D, Fradin S, Ramakers M, Terzi N, Guillotin D, Bouchet B, Daubin C, Charbonneau P: **Angiotensin converting enzyme insertion/deletion genetic polymorphism: its impact on renal function in critically ill patients.** Crit Care Med 2008, **36**(12):3178-3183.

4. Haase-Fielitz A, Haase M, Bellomo R, Lambert G, Matalanis G, Story D, Doolan L, Buxton B, Gutteridge G, Luft FC, Schunck WH, Dragun D: **Decreased catecholamine degradation associates with shock and kidney injury after cardiac surgery.** J Am Soc Nephrol 2009, **20**(6):1393-1403.

5. Jaber BL, Rao M, Guo D, Balakrishnan VS, Perianayagam MC, Freeman RB, Pereira BJ: **Cytokine gene promoter polymorphisms and mortality in acute renal failure.** Cytokine 2004, **25**(5):212-219.

6. Kolyada AY, Tighiouart H, Perianayagam MC, Liangos O, Madias NE, Jaber BL: **A genetic variant of hypoxia-inducible factor-1alpha is associated with adverse outcomes in acute kidney injury.** Kidney Int 2009, **75**(12):1322-1329.

7. Payen D, Lukaszewicz AC, Legrand M, Gayat E, Faivre V, Megarbane B, Azoulay E, Fieux F, Charron D, Loiseau P, Busson M: **A multicentre study of acute kidney injury in severe sepsis and septic shock: association with inflammatory phenotype and HLA genotype.** PLoS ONE 2012, **7**(6):e35838.

8. Pedroso JA, Paskulin D, Dias FS, de Franca E, Alho CS: **Temporal trends in acute renal dysfunction among critically ill patients according to I/D and -262A > T ACE polymorphisms.** J Bras Nefrol 2010, **32**(2):182-194.

9. Perianayagam MC, Liangos O, Kolyada AY, Wald R, MacKinnon RW, Li L, Rao M, Balakrishnan VS, Bonventre JV, Pereira BJ, Jaber BL: **NADPH oxidase p22phox and catalase gene variants are associated with biomarkers of oxidative stress and adverse outcomes in acute renal failure.** J Am Soc Nephrol 2007, **18**(1):255-263.

10. Perianayagam MC, Tighiouart H, Nievergelt CM, O'Connor DT, Liangos O, Jaber BL: **CYBA Gene Polymorphisms and Adverse Outcomes in Acute Kidney Injury: A Prospective Cohort Study.** Nephron Extra 2011, **1**(1):112-123.

11. Perianayagam M, Tighiouart H, Liangos O, Kouznetsov D, Wald R, Rao F, O’Connor D, Jaber B: **Polymorphisms in the myeloperoxidase gene locus are associated with acute kidney injuryrelated outcomes.** Kidney international 2012, **82**:909-919.

12. Popov AF, Hinz J, Schulz EG, Schmitto JD, Wiese CH, Quintel M, Seipelt R, Schoendube FA: **The eNOS 786C/T polymorphism in cardiac surgical patients with cardiopulmonary bypass is associated with renal dysfunction.** Eur J Cardiothorac Surg 2009, **36**(4):651-656.

13. Popov AF, Schulz EG, Schmitto JD, Coskun KO, Tzvetkov MV, Kazmaier S, Zimmermann J, Schondube FA, Quintel M, Hinz J: **Relation between renal dysfunction requiring renal replacement therapy and promoter polymorphism of the erythropoietin gene in cardiac surgery.** Artif Organs 2010, **34**(11):961-968.

14. Susantitaphong P, Perianayagam MC, Tighiouart H, Liangos O, Bonventre JV, Jaber BL: **Tumor necrosis factor alpha promoter polymorphism and severity of acute kidney injury.** Nephron 2013, **123**(1-2):67-73.
