# Supplementary material for: Genetic predisposition to acute kidney injury – a systematic review
Source: BMC Nephrol. 2015 Dec 2;16:197. doi: 10.1186/s12882-015-0190-6 (PMC4667497; doi:10.1186/s12882-015-0190-6)
Supplement: Additional file 6: — Quality scoring of the 28 full articles. (PDF 129 kb) [file 12882_2015_190_MOESM6_ESM.pdf]

Additional file 6. Quality scoring of the 28 full articles.

First author	Pub. year	Quality score 1	Quality score 2	Quality score 3	Quality score 4	Quality score 5	Quality score 6	Quality score 7	Quality score 8	Quality score 9	Quality score 10	Quality score total
**Susceptibility**												
**Albert, C.** [1]	2014	0	1	1	1	1	1	0	1	1	1	**8**
**Boehm, J.** [2]	2014	1	0	1	1	0	0	1	1	0	1	**6**
**Cardinal-Fernández, P.** [3]	2013	1	1	1	1	1	1	0	1	0	1	**8**
**Chang, C.** [4]	2013	1	1	1	1	1	0	0	1	0	0	**6**
**Chew, S.** [5]	2000	0	0	1	1	0	1	0	1	1	0	**5**
**Frank, A.** [6]	2012	1	1	1	1	1	1	1	1	1	1	**10**
**Henao-Martínez, A.** [7]	2013	1	1	0	1	1	0	0	1	1	0	**6**
**Isbir, S.** [8]	2007	0	0	1	1	0	0	0	1	1	1	**5**
**Jouan, J.** [9]	2012	0	1	1	1	0	0	0	1	1	0	**5**
**Kornek, M.** [10]	2013	0	1	1	1	0	0	1	1	1	1	**7**
**MacKensen, G.** [11]	2004	1	0	1	1	0	1	1	1	1	0	**7**
**McBride, W.** [12]	2013	0	0	1	0	0	0	0	1	0	1	**3**
**Stafford-Smith, M.** [13]	2005	1	0	1	1	1	1	0	1	1	1	**8**
**Sole-Violan, J.** [14]	2011	1	1	1	1	0	0	1	1	1	1	**8**
**Susceptibility and outcome**												
**Alam, A.** [15]	2010	1	1	1	0	0	0	0	1	0	0	**4**
**Dalboni, M.** [16]	2013	0	1	1	1	1	0	0	1	0	1	**6**
**du Cheyron, D.** [17]	2008	1	1	1	1	1	1	1	1	1	0	**9**
**Haase-Fielitz, A.** [18]	2009	1	1	1	1	1	1	0	1	1	0	**8**
**Payen, D.** [19]	2012	1	0	1	1	0	0	0	1	0	0	**4**

**Pedroso, J.** [20]	2010	0	1	1	1	0	1	0	1	0	0	**5**
**Popov, A.** [21]	2009	1	1	1	1	0	0	0	1	1	0	**6**
**Popov, A.** [22]	2010	1	1	0	1	1	0	0	1	1	0	**6**
**Outcome**												
**Jaber, B.** [23]	2004	1	0	0	1	0	1	0	1	0	0	**4**
**Kolyada, A.** [24]	2009	1	1	1	1	0	0	0	1	1	0	**6**
**Perianayagam, M.** [25]	2007	1	1	1	1	1	1	0	1	0	0	**7**
**Perianayagam, M.** [26]	2011	1	1	1	1	0	0	0	1	0	0	**5**
**Perianayagam, M.** [27]	2012	1	1	1	1	0	0	0	1	1	1	**7**
**Susantitaphong, P.** [28]	2013	1	1	1	1	1	1	0	1	1	1	**9**
**Average**		0.71	0.71	0.89	0.93	0.43	0.43	0.21	1	0.61	0.43	6.36

References

1. Albert C, Kube J, Haase-Fielitz A, Dittrich A, Schanze D, Zenker M, Kuppe H, Hetzer R, Bellomo R, Mertens PR, Haase M: **Pilot study of association of catechol-O-methyl transferase rs4680 genotypes with acute kidney injury and tubular stress after open heart surgery.** Biomark med 2014, **8**(10):1227-1238.

2. Boehm J, Eichhorn S, Kornek M, Hauner K, Prinzing A, Grammer J, Lahm H, Wagenpfeil S, Lange R: **Apolipoprotein E genotype, TNF-alpha 308G/A and risk for cardiac surgery associated-acute kidney injury in Caucasians.** Ren Fail 2014, **36**(2):237-243.

3. Cardinal-Fernandez P, Ferruelo A, El-Assar M, Santiago C, Gomez-Gallego F, Martin-Pellicer A, Frutos-Vivar F, Penuelas O, Nin N, Esteban A, Lorente JA: **Genetic predisposition to acute kidney injury induced by severe sepsis.** J Crit Care 2013, **28**(4):365-370.

4. Chang CF, Lu TM, Yang WC, Lin SJ, Lin CC, Chung MY: **Gene polymorphisms of interleukin-10 and tumor necrosis factor-alpha are associated with contrast-induced nephropathy.** Am J Nephrol 2013, **37**(2):110-117.

5. Chew ST, Newman MF, White WD, Conlon PJ, Saunders AM, Strittmatter WJ, Landolfo K, Grocott HP, Stafford-Smith M: **Preliminary report on the association of apolipoprotein E polymorphisms, with postoperative peak serum creatinine concentrations in cardiac surgical patients.** Anesthesiology 2000, **93**(2):325-331.

6. Frank AJ, Sheu CC, Zhao Y, Chen F, Su L, Gong MN, Bajwa E, Thompson BT, Christiani DC: **BCL2 genetic variants are associated with acute kidney injury in septic shock*.** Crit Care Med 2012, **40**(7):2116-2123.

7. Henao-Martinez AF, Agler AH, LaFlamme D, Schwartz DA, Yang IV: **Polymorphisms in the SUFU gene are associated with organ injury protection and sepsis severity in patients with Enterobacteriacea bacteremia.** Infect Genet Evol 2013, **16**:386-391.

8. Isbir SC, Tekeli A, Ergen A, Yilmaz H, Ak K, Civelek A, Zeybek U, Arsan S: **Genetic polymorphisms contribute to acute kidney injury after coronary artery bypass grafting.** Heart Surg Forum 2007, **10**(6):E439-44.

9. Jouan J, Golmard L, Benhamouda N, Durrleman N, Golmard JL, Ceccaldi R, Trinquart L, Fabiani JN, Tartour E, Jeunemaitre X, Menasche P: **Gene polymorphisms and cytokine plasma levels as predictive factors of complications after cardiopulmonary bypass.** J Thorac Cardiovasc Surg 2012, **144**(2):467; Aug-473.

10. Kornek M, Deutsch MA, Eichhorn S, Lahm H, Wagenpfeil S, Krane M, Lange R, Boehm J: **COMT-Val158Met-polymorphism is not a risk factor for acute kidney injury after cardiac surgery.** Dis Markers 2013, **35**(2):129-134.

11. MacKensen GB. Swaminathan M. Ti LK. Grocott HP. Phillips-Bute BG. Mathew JP. Newman MF. Milano CA. Stafford-Smith M. Perioperative Outcomes Research Group. Cardiothoracic Anesthesiology Research Endeavors (C.A.R.E.) Investigators of the Duke Heart Center: **Preliminary report on the interaction of apolipoprotein E polymorphism with aortic atherosclerosis and acute nephropathy after CABG.** Ann Thorac Surg 2004, **78**(2):520-526.

12. McBride WT, Prasad PS, Armstrong M, Patterson C, Gilliland H, Drain A, Vuylsteke A, Latimer R, Khalil N, Evans A, Cambien F, Young I: **Cytokine phenotype, genotype, and renal outcomes at cardiac surgery.** Cytokine 2013, **61**(1):275-284.

13. Stafford-Smith M. Podgoreanu M. Swaminathan M. Phillips-Bute B. Mathew JP. Hauser EH. Winn MP. Milano C. Nielsen DM. Smith M. Morris R. Newman MF. Schwinn DA. Perioperative Genetics and Safety Outcomes Study (PEGASUS) Investigative Team: **Association of genetic polymorphisms with risk of renal injury after coronary bypass graft surgery.** Am J Kidney Dis 2005, **45**(3):519-530.

14. Sole-Violan J, Garcia-Laorden MI, Marcos-Ramos JA, de Castro FR, Rajas O, Borderias L, Briones ML, Herrera-Ramos E, Blanquer J, Aspa J, Florido Y, Garcia-Bello MA, Ferrer-Aguero JM, Sologuren I, Rodriguez-Gallego C: **The Fc receptor IIA-H/H131 genotype is associated with bacteremia in pneumococcal community-acquired pneumonia.** Crit Care Med 2011, **39**(6):1388-1393.

15. Alam A, O'Connor DT, Perianayagam MC, Kolyada AY, Chen Y, Rao F, Mahata M, Mahata S, Liangos O, Jaber BL: **Phenylethanolamine N-methyltransferase gene polymorphisms and adverse outcomes in acute kidney injury.** Nephron 2010, **114**(4):253-259.

16. Dalboni MA, Quinto BM, Grabulosa CC, Narciso R, Monte JC, Durao M Jr, Rizzo L, Cendoroglo M, Santos OP, Batista MC: **Tumour necrosis factor-alpha plus interleukin-10 low producer phenotype predicts acute kidney injury and death in intensive care unit patients.** Clin Exp Immunol 2013, **173**(2):242-249.

17. du Cheyron D, Fradin S, Ramakers M, Terzi N, Guillotin D, Bouchet B, Daubin C, Charbonneau P: **Angiotensin converting enzyme insertion/deletion genetic polymorphism: its impact on renal function in critically ill patients.** Crit Care Med 2008, **36**(12):3178-3183.

18. Haase-Fielitz A, Haase M, Bellomo R, Lambert G, Matalanis G, Story D, Doolan L, Buxton B, Gutteridge G, Luft FC, Schunck WH, Dragun D: **Decreased catecholamine degradation associates with shock and kidney injury after cardiac surgery.** J Am Soc Nephrol 2009, **20**(6):1393-1403.

19. Payen D, Lukaszewicz AC, Legrand M, Gayat E, Faivre V, Megarbane B, Azoulay E, Fieux F, Charron D, Loiseau P, Busson M: **A multicentre study of acute kidney injury in severe sepsis and septic shock: association with inflammatory phenotype and HLA genotype.** PLoS ONE 2012, **7**(6):e35838.

20. Pedroso JA, Paskulin D, Dias FS, de Franca E, Alho CS: **Temporal trends in acute renal dysfunction among critically ill patients according to I/D and -262A > T ACE polymorphisms.** J Bras Nefrol 2010, **32**(2):182-194.

21. Popov AF, Hinz J, Schulz EG, Schmitto JD, Wiese CH, Quintel M, Seipelt R, Schoendube FA: **The eNOS 786C/T polymorphism in cardiac surgical patients with cardiopulmonary bypass is associated with renal dysfunction.** Eur J Cardiothorac Surg 2009, **36**(4):651-656.

22. Popov AF, Schulz EG, Schmitto JD, Coskun KO, Tzvetkov MV, Kazmaier S, Zimmermann J, Schondube FA, Quintel M, Hinz J: **Relation between renal dysfunction requiring renal replacement therapy and promoter polymorphism of the erythropoietin gene in cardiac surgery.** Artif Organs 2010, **34**(11):961-968.

23. Jaber BL, Rao M, Guo D, Balakrishnan VS, Perianayagam MC, Freeman RB, Pereira BJ: **Cytokine gene promoter polymorphisms and mortality in acute renal failure.** Cytokine 2004, **25**(5):212-219.

24. Kolyada AY, Tighiouart H, Perianayagam MC, Liangos O, Madias NE, Jaber BL: **A genetic variant of hypoxia-inducible factor-1alpha is associated with adverse outcomes in acute kidney injury.** Kidney Int 2009, **75**(12):1322-1329.

25. Perianayagam MC, Liangos O, Kolyada AY, Wald R, MacKinnon RW, Li L, Rao M, Balakrishnan VS, Bonventre JV, Pereira BJ, Jaber BL: **NADPH oxidase p22phox and catalase gene variants are associated with biomarkers of oxidative stress and adverse outcomes in acute renal failure.** J Am Soc Nephrol 2007, **18**(1):255-263.

26. Perianayagam MC, Tighiouart H, Nievergelt CM, O'Connor DT, Liangos O, Jaber BL: **CYBA Gene Polymorphisms and Adverse Outcomes in Acute Kidney Injury: A Prospective Cohort Study.** Nephron Extra 2011, **1**(1):112-123.

27. Perianayagam M, Tighiouart H, Liangos O, Kouznetsov D, Wald R, Rao F, O'Connor D, Jaber B: **Polymorphisms in the myeloperoxidase gene locus are associated with acute kidney injuryrelated outcomes.** Kidney international 2012, **82**:909-919.

28. Susantitaphong P, Perianayagam MC, Tighiouart H, Liangos O, Bonventre JV, Jaber BL: **Tumor necrosis factor alpha promoter polymorphism and severity of acute kidney injury.** Nephron 2013, **123**(1-2):67-73.
